# How a Realistic Magnetosphere Alters the Polarizations of Surface, Fast Magnetosonic, and Alfvén Waves

**DOI:** 10.1029/2021JA030032

**Published:** 2022-02-07

**Authors:** M. O. Archer, D. J. Southwood, M. D. Hartinger, L. Rastaetter, A. N. Wright

**Affiliations:** ^1^ Space and Atmospheric Physics Group, Department of Physics Imperial College London London UK; ^2^ Space Science Institute Boulder CO USA; ^3^ NASA Goddard Space Flight Center Greenbelt MD USA; ^4^ Department of Mathematics and Statistics University of St Andrews St Andrews UK

**Keywords:** ULF waves, polarization, magnetohydrodynamics, simulation, theory

## Abstract

System‐scale magnetohydrodynamic (MHD) waves within Earth's magnetosphere are often understood theoretically using box models. While these have been highly instructive in understanding many fundamental features of the various wave modes present, they neglect the complexities of geospace such as the inhomogeneities and curvilinear geometries present. Here, we show global MHD simulations of resonant waves impulsively excited by a solar wind pressure pulse. Although many aspects of the surface, fast magnetosonic (cavity/waveguide), and Alfvén modes present agree with the box and axially symmetric dipole models, we find some predictions for large‐scale waves are significantly altered in a realistic magnetosphere. The radial ordering of fast mode turning points and Alfvén resonant locations may be reversed even with monotonic wave speeds. Additional nodes along field lines that are not present in the displacement/velocity occur in both the perpendicular and compressional components of the magnetic field. Close to the magnetopause, the perpendicular oscillations of the magnetic field have the opposite handedness to the velocity. Finally, widely used detection techniques for standing waves, both across and along the field, can fail to identify their presence. We explain how all these features arise from the MHD equations when accounting for a non‐uniform background field and propose modified methods that might be applied to spacecraft observations.

## Key Points

- A global MHD simulation shows system‐scale ULF waves' polarizations can significantly differ from box and dipole model predictions
- Phase or handedness reversals in the magnetic field compared to the velocity can occur simply due to the highly non‐uniform background field
- We propose modified detection techniques for spacecraft observations which account for the effects of a realistic magnetosphere

## 1 Introduction

**Figure 1:**
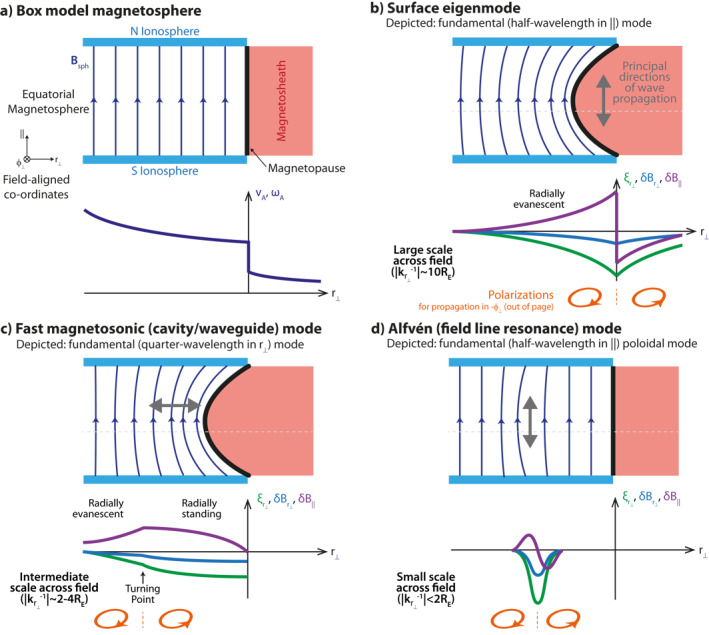
Cartoon illustrating (a) a box model magnetosphere and (b)–(d) MHD wave eigenmodes within it. Shown are a magnetopause surface eigenmode (b), cavity/waveguide mode (c), and poloidal field line resonance (d). Inset graphs show (a) a monotonic Alfvén speed profile, (b)–(d) instantaneous variations along the dotted lines of radial displacement (green) and perpendicular (blue) and compressional (purple) magnetic field perturbations. The sense of the polarisation is also shown in orange, assuming westward (out of the page) propagating disturbances.

System‐scale magnetohydrodynamic (MHD) waves in Earth's magnetosphere are routinely observed by spacecraft and ground‐based instrumentation as ultra‐low frequency (ULF) waves, with frequencies of fractions of milliHertz to a few Hertz (Jacobs et al., 1964). These waves provide a means for solar wind energy and momentum to be transferred throughout geospace, for example, to the radiation belts (Elkington, 2006). They can also, through understanding the various normal modes they may establish, be used as a tool for probing the ever‐changing nature of the terrestrial system (Menk & Waters, 2013). The foundations of MHD wave theory have largely been built in so‐called box models, where the curved geomagnetic field lines are straightened into a uniform field anchored at the northern and southern ionospheres due to their high conductivity as shown in Figure 1a (e.g., Radoski, 1971; Southwood, 1974). While such analytic models have proven extremely useful, they are of course highly simplified compared to reality, thus it is important to understand their limitations. Numerical modeling of MHD waves can help in this regard and there are a number of different approaches which may be taken, from dedicated wave codes (e.g., Degeling et al., 2014; Lee & Lysak, 1999; Wright & Elsden, 2016) to the use of general purpose global simulations (e.g., Claudepierre et al., 2010; Ellington et al., 2016; Hartinger et al., 2014). Wright and Elsden (2020) provide further discussion of the benefits/drawbacks to each approach.

Comparing the polarization with theoretical predictions is an often used method of deciphering the wave modes present within ULF wave observations, both at Earth (e.g., Agapitov et al., 2009; Kokubun, 2013; Mathie et al., 1999; Samson et al., 1971; Takahashi et al., 1991) and other planetary systems (e.g., James et al., 2016; Manners & Masters, 2019). Polarization can refer to several different related aspects of a wave including: the orientation of oscillations of a particular physical quantity, the shape and handedness these trace out, relative amplitudes between different quantities, or their cross‐phases (Waters et al., 2002). When waves have a definite sense of propagation azimuthally (assumed westwards throughout Figure 1), their polarization depends only on the gradient in amplitude across the field, which in typical box and dipole models is either radially toward/away from the Earth, and whether the waves are evanescent/propagating (Southwood, 1974; Southwood & Kivelson, 1986). In the following subsections, we briefly introduce the surface, fast magnetosonic, and Alfvén eigenmodes of the magnetosphere.

### 1.1 Surface Modes

The dynamics of discontinuities in geospace may be described as surface waves driven by upstream pressure variations or flow shears (Kivelson & Chen, 1995; Pu & Kivelson, 1983). These have mostly been studied at the magnetopause flanks, where amplitudes can grow via the Kelvin‐Helmholtz instability (Fairfield et al., 2000; Otto & Fairfield, 2000). Here, the waves' frequency (*ω*/2*π*) is largely controlled by the magnetosheath velocity **v**_*msh*_, that is, $\left|\omega -k\cdot v_{msh}\right|\approx k_{\phi }v_{msh}$ where **k** is the wavevector and *ϕ* the azimuthal direction (see Notation). However, on the dayside, where flows are weaker, the finite extent of magnetospheric field lines play a more significant factor. Chen and Hasegawa (1974) proposed the possibility of surface eigenmodes between conjugate ionospheres, only recently discovered observationally at the magnetopause (Archer et al., 2019) and plasmapause (He et al., 2020). Figure 1b illustrates a magnetopause surface eigenmode (MSE) in a box model (Plaschke & Glassmeier, 2011), constructed as evanescent fast magnetosonic waves either side of an infinitesimally thin discontinuity. Surface waves therefore obey the usual fast wave dispersion relation

(1) $$k_{r_{⊥}}^{2}=-k_{\phi_{⊥}}^{2}-k_{∥}^{2}+\frac{\omega^{4}}{\omega^{2}v_{A}^{2}+c_{s}^{2}\left(\omega^{2}-k_{∥}^{2}v_{A}^{2}\right)}$$

where *v*_*A*_ and *c*_*s*_ are the Alfvén and sound speeds respectively. Incompressibility renders the last term of Equation 1 negligible. The frequency is dictated by conditions either side

(2) $$\omega_{MSE}=k_{∥}\sqrt{\frac{B_{sph}^{2}+B_{msh}^{2}}{\mu_{0}\left(\rho_{sph}+\rho_{msh}\right)}}\approx k_{∥}\frac{B_{sph}}{\sqrt{\mu_{0}\rho_{msh}}}$$

for $k_{\phi_{⊥}}\ll k_{∥}$ (Plaschke et al., 2009), which predicts fundamental frequencies below 2 mHz (Archer et al., 2015). This makes MSE the lowest frequency magnetospheric normal mode and highly penetrating. A finite thickness boundary is thought to damp these collective modes through mode‐conversion to oscillations within the Alfvén speed gradient, which undergo spatial phase‐mixing and dissipate energy to smaller (non‐MHD) scales (Chen & Hasegawa, 1974; Lee & Roberts, 1986; Uberoi, 1989). Whether this all occurs locally or if energy is deposited to the ionosphere is not currently known.

While it is not understood theoretically how more realistic magnetic geometries affect surface waves (Archer & Plaschke, 2015; Kozyreva et al., 2019), high‐resolution global MHD simulations have provided valuable insights. Hartinger et al. (2015), henceforth H15, showed global 1.8 mHz waves excited by a solar wind density pulse, consistent only with MSE. The amplitude of the magnetic compressions/rarefactions decay with distance from the magnetopause. However, inside the current layer two local maxima occur, with a minimum between them near the peak current density. The phase of the compressional magnetic field reverses either side of the boundary, that is, when the magnetosphere is compressed the magnetosheath becomes rarefied. Archer et al. (2021), A21 herein, found similar motion of the subsolar bow shock, lagging behind the magnetopause. While the magnetopause waves travel tailward at the equatorial flanks, between 09 and 15 hr Magnetic Local Time (MLT) they are stationary despite significant magnetosheath flows being present. The authors show the time‐averaged Poynting flux inside the magnetosphere surprisingly points toward the subsolar point, perfectly balancing advection by the magnetosheath flow such that there is no net (Poynting plus advective) flux. Inside the magnetosphere, despite decaying amplitudes with distance, phase fronts slowly propagate toward the magnetopause due to damping. Finally, the Kelvin‐Helmholtz instability causes seeded tailward propagating surface waves to grow in amplitude.

### 1.2 Fast Magnetosonic (Cavity/Waveguide) Modes

Fast magnetosonic waves may form radially standing waves due to reflection by boundaries (such as the magnetopause) or turning points (where $k_{r_{⊥}}$ from Equation 1 becomes zero; Kivelson & Southwood, 1985; Kivelson et al., 1984). These are known as cavity modes in closed geometries (Allan et al., 1986) or waveguides when the magnetosphere is open‐ended (Samson et al., 1992; Wright, 1994). Azimuthal wavenumbers are thus continuous in the latter but quantized in the former. Many types of cavity/waveguide modes are known such as plasmaspheric, virtual, tunneling, and trapped modes (Waters et al., 2000), with Figure 1c depicting one between the magnetopause and a turning point, beyond which the wave is evanescent.

The fast eigenmodes can be estimated under the Wentzel‐Kramers‐Brillouin (WKB) approximation (full numerical solutions differ by only ∼3%; Rickard & Wright, 1995) by spatially integrating the radial wavenumber (Equation 1) and imposing a quantisation condition (Samson et al., 1992, 1995). The fundamental mode was originally thought to be a half‐wavelength mode, with a node in displacement at the magnetopause (Kivelson & Southwood, 1985; Samson et al., 1992). Mann et al. (1999) later showed that quarter‐wavelength modes with a displacement antinode at the boundary may be possible, which is what is shown in Figure 1c. Both these modes have been successfully reproduced around noon within global MHD simulations (Claudepierre et al., 2009; Hartinger et al., 2014). Cavity/waveguide modes' structure and frequencies are thought to be highly dependent on the Alfvén speed profiles present (Allan & McDiarmid, 1989; Archer et al., 2015, 2017; Wright & Rickard, 1995), though in general have higher frequencies and less penetrating scales than surface modes. While numerical works suggest they should have clear compressional magnetic field signatures with nodal structure radially (Elsden & Wright, 2018, 2019; Waters et al., 2002), identifying them in satellite observations can be challenging (Hartinger et al., 2012, 2013).

### 1.3 Alfvén Modes

The final mode concerns Alfvén waves standing along geomagnetic field lines (Dungey, 1967). Often these occur over a range of *L*‐shells with a continuum of resonant frequencies present, however, sometimes a discrete field line resonance is established (e.g., Plaschke et al., 2008), as depicted in Figure 1d. Alfvén modes are typically described in terms of either poloidal or toroidal polarization. In an axially symmetric dipole, the toroidal mode corresponds to azimuthal displacements of the plasma (and thus do not lead to magnetic compressions) whereas poloidal modes feature radial ones (we note compressions become negligible for high azimuthal wavenumbers though). WKB methods predict no difference in frequencies between the two orientations, with the fundamental given by

(3) $$\omega_{A}\approx 2\pi {\left[2\int \frac{ds}{v_{A}}\right]}^{-1}$$

Singer et al. (1981), however, derived the wave equation within a general orthogonal magnetic geometry

(4) $$\frac{\partial^{2}}{\partial s^{2}}\left(\frac{\xi_{\alpha }}{h_{\alpha }}\right)+\frac{\partial }{\partial s}\left(\ln\left[h_{\alpha }^{2}B_{0}\right]\right)\frac{\partial }{\partial s}\left(\frac{\xi_{\alpha }}{h_{\alpha }}\right)+\frac{\omega^{2}}{v_{A}^{2}}\left(\frac{\xi_{\alpha }}{h_{\alpha }}\right)=0$$

where *α* represents some direction perpendicular to the background field and *h*_*α*_ is its corresponding scale factor, estimated as the distance between adjacent field lines. Numerical solutions to this equation predict lower frequencies for the poloidal mode than the toroidal one, which have been verified within simulations (e.g., Elsden & Wright, 2020). However, orthogonal coordinates only exist in the absence of background field‐aligned currents (Salat & Tataronis, 2000) and improvements that do not require an orthogonal system have also been developed (Degeling et al., 2010; Kabin et al., 2007; Rankin et al., 2006). These have shown that the orientations of the two polarizations can be altered by the local magnetic geometry. Asymmetries in the Alfvén speeds can also have a similar effect (Wright & Elsden, 2020). Field line resonances can also be reproduced in global MHD simulations (Claudepierre et al., 2010; Ellington et al., 2016). The width of a discrete field line resonance is given by the length scale of radial changes in the eigenfrequency (Mann et al., 1995; Southwood & Allan, 1987), which typically gives much shorter scales than the other two modes.

### 1.4 Preface

The three wave modes do not exist in isolation. In box and axially symmetric dipole model setups wave coupling depends on the azimuthal wavenumber, with no coupling predicted in the limits of zero or infinity (Chen & Cowley, 1989; Kivelson & Southwood, 1985). However, in more realistic geometries coupling is always expected (Radoski, 1971). Typically this is discussed as the surface (Southwood, 1974) or cavity/waveguide (Kivelson & Southwood, 1985; Kivelson et al., 1984) mode exciting a field line resonance at the radial location where their eigenfrequencies match. This theory, however, is usually one‐dimensional in nature and the problem of wave coupling in a 3D asymmetric magnetosphere remains a topic of current research. While dedicated MHD wave codes have shown progress in the area of fast‐Alfvén mode coupling, only global MHD simulations can self‐consistently incorporate magnetopause surface modes too. Therefore, in this study, we study one such simulation run to determine how wave polarizations may be altered in a realistic magnetosphere compared to the simplified box models.

## 2 Simulation

This study uses a high‐resolution Space Weather Modeling Framework (SWMF; Tóth et al., 2005, 2012) simulation of the magnetospheric response to a 1‐min solar wind density pulse with sunward normal, pressure‐balanced with the ambient plasma via reduced temperature. The interplanetary magnetic field (IMF) is kept constant and northward, since this is most conducive to surface eigenmodes and the Kelvin‐Helmholtz instability (Hasegawa, 1975; Plaschke & Glassmeier, 2011; Southwood, 1968). No plasmasphere or ring current is included. The ionospheric conductivity is uniform and the dipole is fixed with zero tilt throughout. The simulation hence is both North‐South and dawn‐dusk symmetric. Full details of the parameters used are found in Table S1. This specific simulation run was first presented by A21 which in turn was essentially a replication on NASA's Community Coordinated Modeling Center (CCMC) of the simulation originally described by H15. We only use the BATS‐R‐US (Block‐Adaptive‐Tree‐Solarwind‐Roe‐Upwind‐Scheme) global MHD results here, leaving the other regions covered by SWMF, such as the ionosphere and ground magnetometer response, to potential future work. We focus on the dayside and near flanks of the magnetosphere (*X*_*GSM*_ > −15 *R*_*E*_) for which the grid‐resolution of the simulation is 1/8 *R*_*E*_ throughout, apart from around the inner boundary where a 1/16 *R*_*E*_ resolution shell is used between 2.5 and 5 *R*_*E*_ geocentric distance.

A proxy for the magnetopause location is used within the CCMC tools, given by the last closed field line along geocentric rays through a bisection method accurate to 0.01 *R*_*E*_ (fields are interpolated in the tracing). Throughout, perturbations (represented by *δ*’s) from the background (represented by subscript 0's) are defined as the difference to the linear trend. We focus on the resonant response following the driving phase, that is, neglecting the transient wave activity directly driven by the pulse. This is done by expressing the time that the magnetopause returns to equilibrium as a function of *X*_*GSM*_, extending this throughout the grid, and then adding half the lowest wave period present in the boundary motion (A21). Vector quantities are rotated into local field‐aligned coordinates where the field‐aligned direction **e**_∥_ points along the time‐average of the background magnetic field, the (perpendicular) azimuthal direction $e_{\phi_{⊥}}=\left(e_{∥}\times r\right)/\left|e_{∥}\times r\right|$ points eastwards (**r** is the geocentric position), and the (perpendicular) radial direction $e_{r_{⊥}}=e_{\phi_{⊥}}\times e_{∥}$ is directed outwards (note this exhibits a discontinuity along the center of the cusps due to a reversal of direction either side).

## 3 Results

At each grid point, we compute power spectra of the traces (sums over all components) of the velocity and magnetic fields, respectively. Spectral peaks whose prominence (how much the peak stands out from the surrounding baseline) is greater than the two‐tailed 95% confidence interval of the spectral estimator have been identified. These reveal spikes in occurrence for both physical quantities at 1.8, 3.1, 6.8, and 11.7 mHz, with the lowest frequency being ∼2–8 times more prevalent than the others. While frequencies above 5 mHz were not discussed by A21, the authors showed that the lowest of these frequencies originates at the subsolar magnetopause as MSE whereas the 3.1 mHz frequency corresponds to intrinsic Kelvin‐Helmholtz waves at the flanks (peak frequencies vary with local time from 2.5 to 3.3 mHz but only the higher frequencies are prominent). Throughout this study, we focus on the most widespread frequency of 1.8 mHz, though we discuss how the results may be generalised to different frequencies and/or spatial scales.

### 3.1 Wave Amplitudes and Phases

**Figure 2:**
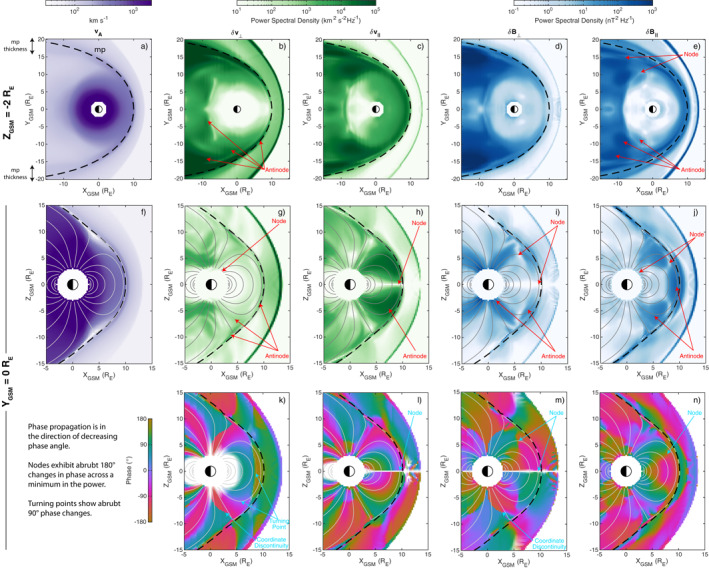
Maps of Alfvén speed (a, f), wave power spectral density (b–e, g–j), and wave phase (k–n) throughout slices of the simulation (arranged by rows) applied to parallel and both perpendicular components of the velocity and magnetic field (arranged by columns). The equilibrium magnetopause location is indicated by the dashed line. Areas of low power are colored white in panels (k–n).

Figure 2 shows maps of average power spectral densities and phases across the 1.0–2.1 mHz frequency band from a Fourier transform of the response phase data. Panels a–e show power in the *Z*_*GSM*_ = −2 *R*_*E*_ plane, though other near‐equatorial slices proved similar. We do not show phases here since there is an ambiguity over which perpendicular directions are most appropriate. Panels f–n show results for the noon meridian, where azimuthal velocities and magnetic fields are zero by symmetry, with Movie S1 also showing bandpass filtered results in this plane (unfiltered movies were presented in A21). To reduce edge effects in the filtering, the first local maxima/minima in the response phase is located at each point, with the data being mirrored before this. A minimum‐order infinite impulse response filter with passband range of 1.0–2.6 mHz was applied in both the forward and reverse directions (zero‐phase).

#### 3.1.1 Near‐Equatorial Planes

The distribution of Alfvén speeds near the equatorial plane, as shown in Figure 2a, has approximate axial symmetry at *L*‐shells less than ∼7 *R*_*E*_, where the relative variation with local time is below 8%, however asymmetries rapidly increase (coefficients of variation up to ∼50%) with radial distance beyond this point. The simulation does not include a plasmasphere (cf. Claudepierre et al., 2016), and thus wave speeds are monotonic with geocentric distance. The thickness of the magnetopause varies significantly with local time, evident in the Alfvén speed as a clear enhancement around the last closed field lines (dashed black line) on the nightside, corresponding well with the current density. This is ∼5 *R*_*E*_ thick at *X*_*GSM*_ = −15 *R*_*E*_ though becomes thinner as you go toward the dayside. While determining the magnetopause thickness from the Alfvén speed is less obvious across the dayside, continuing the locus of points from the nightside to the subsolar point gives good agreement with the thickness of the current layer (9.25 *R*_*E*_ < *X*_*GSM*_ < 10.75 *R*_*E*_ as shown by H15).

Velocity and magnetic field perturbations in the near‐equatorial magnetosphere (panels b–e) are generally strongest nearer the magnetopause and decay with distance from the boundary. There is a clear trend in power with local time also, being weakest around noon and increasing as you go further tailward. As noted by A21, this is due to wave growth of the existing surface modes by the Kelvin‐Helmholtz instability, which can subsequently couple to other modes (Kivelson & Southwood, 1985; Pu & Kivelson, 1983; Southwood, 1974). Parallel velocities (panel c) are generally stronger than perpendicular (the total power across both perpendicular components is shown) ones, indicating wavenumbers $k_{\phi_{⊥}}\ll k_{∥}$ which is generally the case for dayside surface eigenmodes (Plaschke & Glassmeier, 2011; A21). The opposite scenario, present only near the nightside magnetopause, is more typical for tailward traveling surface waves or fast magnetosonic waveguide modes (Mann et al., 1999; Pu & Kivelson, 1983). The dayside response is also predominantly compressional (panel e) since the surface eigenmode is sustained by pressure imbalances across the boundary (Plaschke & Glassmeier, 2011). In contrast, it is the transverse disturbance of the boundary that is of primary importance in Kelvin‐Helmholtz waves (Hasegawa, 1975; Southwood, 1968), hence why perpendicular magnetic field perturbations (panel d) become larger on the nightside. Finally, nodal structure across the field is present. *δ* **v**_⊥_ exhibits subtle antinodes highlighted in panel b. These correspond to surface waves (at/near the magnetopause) or Alfvén modes (deeper in the magnetosphere). *δB*_∥_ has several antinodes (peaks) and nodes (troughs) also, indicative of waveguide modes (Waters et al., 2002). Their alignment is not purely radial, as expected in a symmetric setup, instead appearing to vary with position — furthest downtail and closest to the magnetopause they seem to be standing in approximately ±*Y*_*GSM*_, whereas deeper into the magnetosphere and closer to Earth their normals become more radially oriented. These agree with the gradients of the reciprocal Alfvén speed and thus the refraction of fast waves (Elsden & Wright, 2019; Wright et al., 2018).

#### 3.1.2 Noon Meridian

Figure 2f, showing a cut in the noon meridian, indicates the simulation magnetic field becomes highly non‐dipolar for field lines with high latitude footpoints, due to the presence of the magnetopause and cusps. This, along with the accumulation of plasma in the exterior cusp regions, then affects the Alfvén speed map shown—for instance there are clear decreases in the cusp regions.

**Figure 3:**
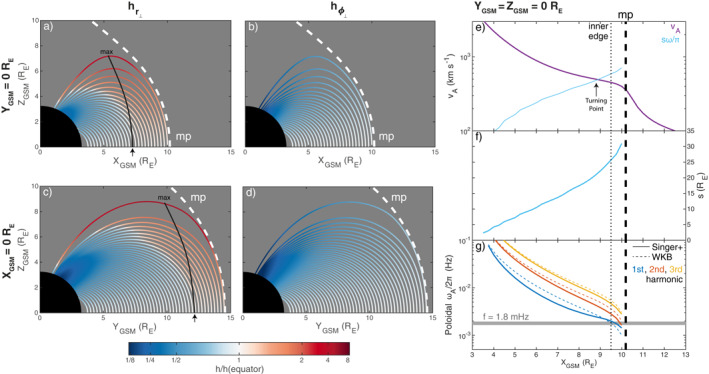
Projected field line tracings in the noon (a–b) and dusk (c–d) meridians colored by the radial (a, c) and azimuthal (b, d) scale factors, normalized by their equatorial value. Scale factor local maxima are indicated by the black line. Also shown are cuts along the subsolar line of the Alfvén speed (e), field line length (f), and estimated first three harmonics of poloidal Alfvén modes (g) using WKB (dotted) and Singer et al. (1981, solid) methods.

The phase of the radial velocity perturbations (Figure 2k) has a sharp 90° shift, indicative of a turning point (Samson et al., 1992; Rickard & Wright, 1994), Earthward of the magnetopause inner edge (at *X*_*GSM*_ = 8.75 *R*_*E*_ on the equator). To understand this theoretically, first, we trace field lines along the subsolar line and compute radial scale factors using the Singer et al. (1981) method (valid here as little reconnection present means background field‐aligned currents are minimal; Stern, 1970; Salat & Tataronis, 2000), resulting in Figure 3a. From these, we can compute field line lengths *s* (panel f) revealing the turning point occurs when the Alfvén speed (panel e) equals the observed wave frequency times by twice the field line length (blue line). This is as expected for a fast mode in cold plasma with zero azimuthal wavenumber and fundamental standing structure along the field (Equation 1). However, the local frequencies of poloidal Alfvén modes in the magnetosphere (computed using both the WKB and Singer et al., 1981, methods as displayed in panel g) are higher than that observed. In fact, the Alfvén frequency only becomes as low as 1.8 mHz on the closed field lines within the magnetopause boundary itself. Kozyreva et al. (2019) suggested that resonant coupling between surface (which is large scale across the field, as observed in the simulation) and Alfvén (which has smaller transverse scales) modes might occur within the transition layer between magnetosheath and magnetospheric plasmas, with this coupling potentially providing a means for surface modes on a boundary of finite thickness to dissipate energy (Chen & Hasegawa, 1974; Lee & Roberts, 1986; Uberoi, 1989). Therefore, despite the Alfvén speed and frequency profiles being monotonic with distance from the magnetopause, we find that surface modes can have turning points which are external to the boundary and thus the usual expected ordering of turning points and resonance locations does not always hold in a realistic magnetosphere. The result should generalize to higher harmonic surface modes since *ω*_*MSE*_ ∝ *k*_∥_ (Equation 2). Similar effects were discussed by Southwood and Kivelson (1986) in a box model magnetosphere with inhomogeneities along the field as well as transverse to it, again even if profiles are monotonic.

**Figure 4:**
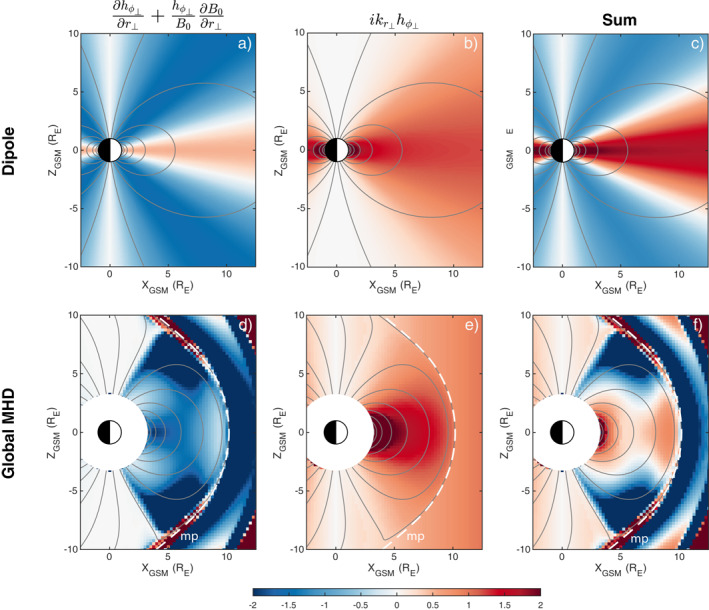
Terms affecting the compressional magnetic field perturbations from Equation 6 dependent on the background field/geometry (a, d) and the wave (b, e), as well as their sum (c, f). These are applied to both a dipole field (a)–(c) and that from the global MHD simulation (d)–(f) for the *Y*_*GSM*_ = 0 *R*_*E*_ plane.

Abrupt amplitude and phase structure are also present along the field too. The parallel velocity, shown in panels h and l of Figure 2, has a node (minimum in power and reversal of phase) at the equator with the phase being relatively constant along field lines either side. The perpendicular velocity (panel k), however, is roughly in‐phase all along each field line down to the inner boundary, that is, there are no nodes present along the field. Both these points are also evident in Movie S1 (middle and right). This standing structure is in agreement with a fundamental surface eigenmode (Figure 1b; Plaschke & Glassmeier, 2011). However, one would expect the perpendicular magnetic field to have antinodes only at the ionospheres with a single node located at the magnetic equator. Instead, additional nodes can be seen at the apexes of the outermost field lines near the cusps (Figure 2m). These can be intuitively understood as being due to the magnetic field geometry—any perpendicular radial displacement that is large‐scale (of order *s*) along the field will not cause deflection of the magnetic field vector here due to the more rapid geometry changes, hence the location is a node in *δ* **B**_⊥_. Similarly, box models predict only one antinode in the compressional magnetic field, also at the equator, with nodes only at the ionospheres. Instead it appears that there are additional antinodes at high latitudes near the cusps that are in antiphase with that at the equator, which can be seen in both Figures 2j and 2n and Movie S1 (left). This might be expected for either a third harmonic mode or if the cusps act to bound the surface mode due to the field's curved geometry, as suggested by Kozyreva et al. (2019). However, we know that at this frequency the velocity exhibits fundamental structure between the conjugate ionospheres. These compressional features, therefore, might be a result of the non‐uniform background field too, but are less intuitive to understand. From the MHD induction equation, the parallel magnetic field is dictated by

(5) $$\begin{array}{ccc} \frac{\partial \delta B_{∥}}{\partial t} & = & -\nabla \cdot \left(B_{0}v_{⊥}\right)\\ & = & -\frac{1}{h_{\alpha }h_{\beta }}\left[\frac{\partial }{\partial \alpha }\left(h_{\beta }B_{0}\delta v_{\alpha }\right)+\frac{\partial }{\partial \beta }\left(h_{\alpha }B_{0}\delta v_{\beta }\right)\right] \end{array}$$

where *β* represents a direction perpendicular to both the background field and *α*. Noting that **v**_⊥_ = *∂* ***ξ***_⊥_/*∂t*, Equation 5 may be expressed throughout the noon meridian, assuming for simplicity a plane wave in the *r*_⊥_ and *ϕ*_⊥_ coordinates, as

(6) $$\begin{array}{ccc} \delta B_{∥} & = & -\nabla \cdot \left(B_{0}\xi_{⊥}\right)\\ & \approx & -\frac{B_{0}}{h_{r_{⊥}}h_{\phi_{⊥}}}\left[\left(\frac{\partial h_{\phi_{⊥}}}{\partial r_{⊥}}+\frac{h_{\phi_{⊥}}}{B_{0}}\frac{\partial B_{0}}{\partial r_{⊥}}+ik_{r_{⊥}}h_{\phi_{⊥}}\right)\xi_{r_{⊥}}+\left(\frac{\partial h_{r_{⊥}}}{\partial \phi_{⊥}}+\frac{h_{r_{⊥}}}{B_{0}}\frac{\partial B_{0}}{\partial \phi_{⊥}}+ik_{\phi_{⊥}}h_{\phi_{⊥}}\right)\xi_{\phi_{⊥}}\right]\\ & \approx & -\frac{B_{0}}{h_{r_{⊥}}h_{\phi_{⊥}}}\left(\frac{\partial h_{\phi_{⊥}}}{\partial r_{⊥}}+\frac{h_{\phi_{⊥}}}{B_{0}}\frac{\partial B_{0}}{\partial r_{⊥}}+ik_{r_{⊥}}h_{\phi_{⊥}}\right)\xi_{r_{⊥}} \end{array}$$

since in our simulation we have $\xi_{\phi_{⊥}}=0$ by symmetry here. The first two terms depend only on the background field and its geometry whereas the third term is largely dictated by the wave itself. The first two terms can be evaluated analytically for a dipole field, as detailed in Appendix A, to give Figure 4a. For the MHD simulation we use the scale factors from the Singer et al. (1981) method shown in Figures 3a and 3b along with the fact that $\partial a/\partial r_{⊥}=h_{r_{⊥}}e_{r_{⊥}}\cdot \left(\nabla a\right)$ for derivatives to arrive at Figure 4d. The radial wavenumber is taken to be $k_{r_{⊥}}\approx -ik_{∥}\approx -i\pi /s$ in panels b and e, which is true for a fundamental surface eigenmode under the assumptions of no damping and incompressibility (Plaschke & Glassmeier, 2011) (for open field lines in the simulation we keep *s* fixed as that of the last closed field line). From the sum of all three terms in Equation 6, both dipole (panel c) and MHD (panel f) fields predict in the high latitude magnetosphere a reversal in sign of the proportionality constant between the compressional magnetic field and the perpendicular displacement. Thus a fundamental mode yields additional nodes and antinodes in the compressional magnetic field. In fact, for the MHD simulation, along the outermost closed field lines (where plasma displacements are largest) there is excellent agreement in the patterns present in the observed compressional perturbations (Figures 2j and 2n) and the predictions based on Equation 6 (Figure 4f). Therefore, a realistic magnetic field can introduce additional structure to the compressional magnetic field oscillations associated with surface modes that are not predicted by box models. As the lowest frequency dayside normal modes of the magnetosphere though, surface eigenmodes have the smallest radial wavenumbers and, following Equation 6, are perhaps most affected by geometrical effects. It is therefore instructive to consider back‐of‐the‐envelope calculations for the other MHD wavemodes. We predict, given the values shown in Figure 4d, that most cavity/waveguide modes and some poloidal Alfvén waves should be altered by the non‐uniform magnetic field—only those with short radial extents $\left(k_{r_{⊥}}^{-1}\ll 1\text{–}2\;R_{E}\right)$ ought to be relatively unaffected. We leave testing these to future work.

Figure 2k–2n indicates, via the gradual decreases in phase radially, the slow perpendicular phase motion discussed by A21 as a result of the surface mode damping. This is even more evident in Movie S1. The movie also reveals interesting behavior at the magnetospheric cusps. As the outermost traced magnetospheric field line shown is displaced by the surface mode, as indicated by the radial velocity, these perturbations clearly propagate through the cusps away from the magnetosphere. Rather than being evanescent, the disturbances appear not to decay in amplitude with distance here, which is backed up by the presence of another turning point at the location of the outermost magnetospheric field line as indicated in Figure 2k. Similar propagating behavior is also seen for the compressional magnetic field at the cusps in Movie S1. While surface modes are often treated theoretically under the assumption of incompressibility, which gives evanescent behavior on both sides of the boundary, the plasma in the exterior cusps is similar to that in the magnetosheath and thus highly compressible (Archer & Plaschke, 2015). This fact predicts (via Equation 1) propagating rather than evanescent magnetosonic waves, as mentioned by A21 in explaining why the subsolar bow shock motion lags the magnetopause by the fast magnetosonic travel time.

### 3.2 Polarization Ellipses

We now investigate the polarizations of the oscillations present throughout the magnetosphere. The orientation and ellipticity parameters of the polarization ellipse as well as the degree of polarization are calculated as detailed in Appendix B for the perpendicular perturbations in the velocity and magnetic field.

#### 3.2.1 Velocity Polarization

**Figure 5:**
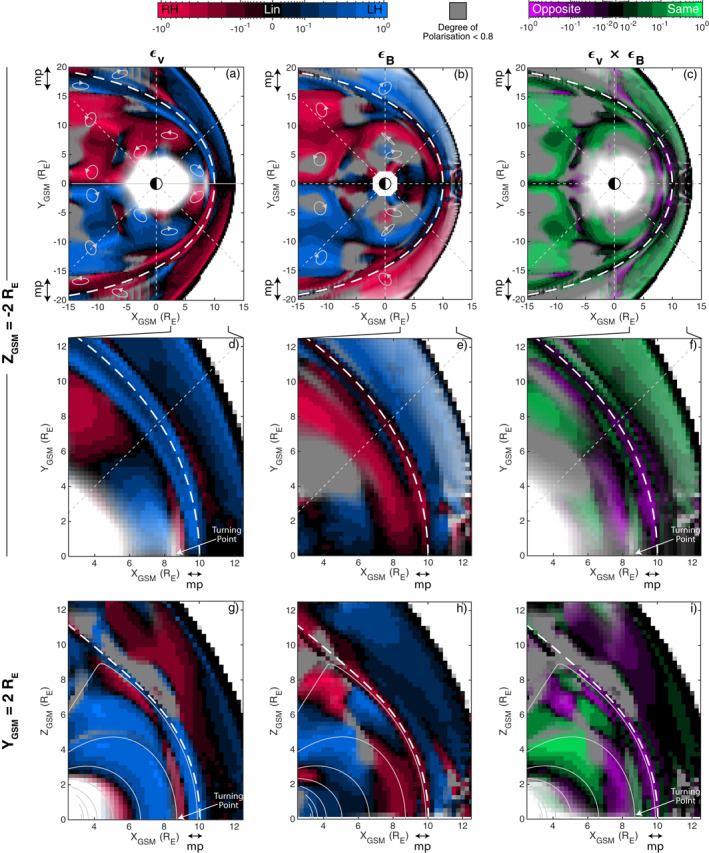
Polarization ellipticities in simulation slices shown for the velocity (a, d, g) and magnetic field (b, e, h). Their product is also shown (c, f, i). Color scales use a bi‐symmetric log transform (Webber, 2012). Regions with low degree of polarization and power are colored gray and white respectively.

**Figure 6:**
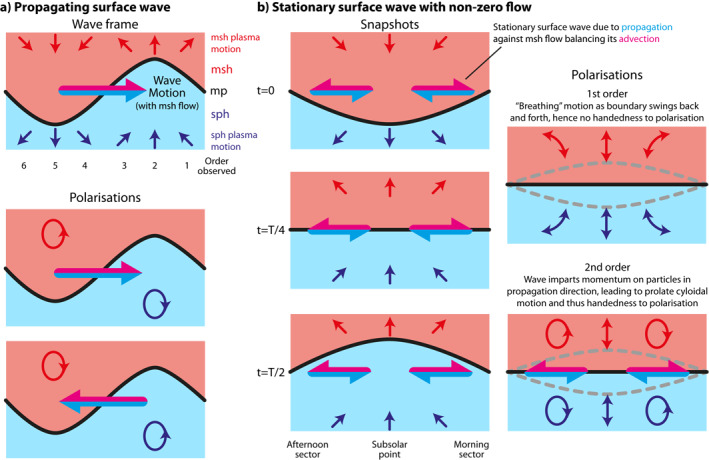
Illustration of the expected velocity polarizations for a propagating (a) and stationary (b) magnetopause surface wave. Plasma motion arrows indicate the displacement that parcel undertook in the last quarter cycle (*T*/4).

Figure 5a shows ellipticities of the velocity in the *Z*_*GSM*_ = −2 *R*_*E*_ plane. These are antisymmetric about the noon‐midnight meridian, with opposite handedness either side, due to the symmetry of the simulation. Throughout the magnetosphere for local times before 09 hr and after 15 hr the polarizations are largely left‐handed and right‐handed with respect to the magnetic field respectively. A21 showed that the magnetopause perturbations propagate tailward at these local times. The results agree with expectations for this scenario, as illustrated in Figure 6a by showing the plasma displacement either side of the boundary in the frame of a surface wave (top) and how this results in a sense of rotation in the Earth's frame as the wave propagates tailward with the magnetosheath flow (bottom; Samson et al., 1971; Southwood, 1968; Stokes, 1847). Either side of noon, the perturbations within the magnetopause current layer are right‐handed on the dawn‐side and left‐handed on the dusk‐side. These are consistent with the Lee et al. (1981) model of surface waves in a boundary layer of finite thickness, where the polarization inside the transition layer is dominated by the mode at the interface with the magnetosheath rather than that with the magnetosphere.

Between 09 and 15 hr MLT, it was shown by A21 that the magnetopause is a stationary wave, with this being achieved by the surface waves propagating against and perfectly balancing the tailward magnetosheath flow. Figure 5d shows a zoom in of the dayside post‐noon sector. This reveals between noon and 15 hr local time that the handedness of the velocity perturbations are mostly left‐handed with respect to the magnetic field, the opposite of that found later in the afternoon. An exception occurs between the inner edge of the magnetopause and the turning point identified previously, where right‐handed waves are present. Figure 5g shows these polarizations remain consistent along the field also. To understand the polarization Earthward of the turning point, where the waves are evanescent, we show snapshots in time of a stationary surface wave in Figure 6b. To first order, a stationary surface wave has no handedness to its polarization, as shown in the top right image of Figure 6b, since the boundary undergoes a simple breathing motion (e.g., Lamb, 1895). However, this prediction neglects any background flow or wave propagation. Stokes (1847) showed by taking into account the evanescent nature of a traveling surface wave's flow patterns, fluid elements' paths are no longer perfect orbits but become cycloidal having moved greater distance in the direction of propagation closer to the boundary than in the opposite direction when farther away (see also Southwood & Kivelson, 1993). Therefore, a surface wave imparts momentum on the particles in the direction of propagation. Since in a stationary magnetopause surface wave subject to non‐zero magnetosheath flow the wave propagates toward the subsolar point (A21), momentum will thus be imparted on the plasma in the same sense. This results in a handedness to the polarization, as depicted in bottom right panel of Figure 6b, which is in agreement with the simulation. The handedness of velocity perturbations in the magnetosphere could thus be used to infer stationary surface waves in spacecraft observations. Between the turning point and the magnetopause the waves are not evanescent, thus a reversal in polarization is expected (Southwood, 1974).

Since Samson et al. (1971), observations have predominantly reported a reversal of ULF wave polarizations only around noon (e.g., Mathie et al., 1999; Ziesolleck & McDiarmid, 1995), conforming with the expectations of tailward propagating disturbances. The polarization patterns presented here thus appear to differ to the typical pattern, though we note other exceptions are reported in the literature. Of note is the recent work of Huang (2021), who presents a case study of global 10 min period waves observed by ∼180 ground magnetometers following a solar wind pressure pulse that is consistent with our simulation results. The author found that the waves (whose period was independent of latitude/longitude and amplitudes increased with latitude) had rotating equivalent currents in the northern hemisphere which were clockwise (i.e., right‐handed with respect to the field) in the morning and evening sectors, and anticlockwise (i.e., left‐handed with respect to the field) in the post‐midnight and afternoon sectors—in agreement with the polarizations presented here. Based on our results, we therefore interpret these observations as due to MSE excited by the impulse.

The projected orientation of the velocity polarization ellipses is also shown in a few locations in Figure 5a. Near the Earth, the semi‐major axes are aligned predominantly in the azimuthal direction, though there is a non‐negligible radial component also (e.g., around noon the polarisation is almost entirely radial). In the nightside (*X*_*GSM*_ < −5 *R*_*E*_) the ellipses tend to be aligned more to lines of constant *X*_*GSM*_. The simulation, therefore, reproduces the fact that a realistic magnetosphere changes the directions of MHD waves' velocity (or equivalently electric field) oscillations compared to those predicted by models with perfect cylindrical symmetry (Degeling et al., 2014; Kabin et al., 2007; Rankin et al., 2006; Wright & Elsden, 2020). Despite the waves present being predominantly compressional, these orientations are approximately perpendicular to the gradient in amplitude as expected for non‐compressional modes rather than parallel to it (Southwood & Kivelson, 1984). In spacecraft observations this has been regularly observed and interpreted as evidence of (non‐resonant) wave coupling between fast and Alfvénic modes.

#### 3.2.2 Magnetic Field Polarization

The same polarization analysis is shown for the magnetic field perturbations in Figures 5b, 5e, and 5h. In a uniform background field, Alfvén’s frozen‐in theorem predicts these should have the same sense as the velocity. Therefore in panels c, f, and i, we also show the product of the two ellipticities, which indicate regions where they are the same (green) or opposite (purple). Throughout most of the near‐equatorial slice they indeed have the same handedness. However, the zoom in on the dayside (panel f) highlights a sizable region within the magnetosphere where their polarizations are opposite (while there are some other instances, these mostly occur within boundary layers or the cusps). Along the subsolar line this region extends from the turning point to *X*_*GSM*_ = 7.5 *R*_*E*_. Panel i shows that this opposite polarization does not extend all the way along the field lines, terminating at some point that extends further in *Z*_*GSM*_ for larger *L*‐shells. This means that near the inner boundary of the simulation the magnetic field returns to having the same handedness as the velocity.

To the best of our knowledge, opposite handedness in the polarizations of velocity and magnetic field oscillations has not been reported before. It is likely that this is due to the non‐uniform background field. Singer et al. (1981) show that the magnetic perturbations in an arbitrary orthogonal field geometry are related to the displacement via

(7) $$\delta B_{\alpha }=h_{\alpha }B_{0}\frac{\partial }{\partial s}\left(\frac{\xi_{\alpha }}{h_{\alpha }}\right)\text{,}$$

valid here due to little shearing being present (Stern, 1970; Salat & Tataronis, 2000). In Figures 3a and 3b, we show how the radial and azimuthal scale factors vary along each field line in the noon meridian. It is clear that the azimuthal scale factors decrease in value along the field either side of the equator. This is also true of the radial scale factors for low *L*‐shells, as expected for an approximately dipolar field (see Appendix A). However, from *X*_*GSM*_ ≥ 7.5 *R*_*E*_ (indicated by the black arrow) $h_{r_{⊥}}$ increases with distance along the field from the equator reaching a maximum as field lines become further apart toward the cusps. The locus of these local maxima are depicted by the black line in panel a. $h_{r_{⊥}}$ changes by up to 4–5× its equatorial value over a fraction of the field line length (∼5%–30%), whereas the scale length along the field of the displacement is 2*s* (a fundamental mode). Therefore, from Equation 7, one would expect geometric effects to dominate as we are in the long wavelength limit. Since *∂h*/*∂s* has opposite signs for the radial and azimuthal directions in the region to the right of the black line, this should lead to a reversal in handedness of the magnetic polarisation compared to the displacement (and thus also the velocity). This region agrees extremely well with the polarizations observed in the simulation, with Figure 5i clearly showing a matching trend with *Z*_*GSM*_. We look for similar evidence of a reversal of handedness along the terminator also, with Figures 3c and 3d showing the traced field lines and scale factors. These predict a reversal at *Y*_*GSM*_ = ±12.25 *R*_*E*_. Figure 5b does indeed show a reversal in the handedness of the magnetic field around these points. Unfortunately though the velocity perturbations in this region are almost linearly polarized and thus it is unclear whether the magnetic field and velocity are of opposite polarization here. Nonetheless, our results highlight that care needs to be taken when using the polarization of the magnetic field from spacecraft observations (e.g., Agapitov et al., 2009; Kokubun, 2013; Takahashi et al., 1991), as close to the magnetopause this can be reversed with respect to the displacement/velocity purely due to the highly curvilinear geometry present and likely affects many developed ULF wave diagnostics based on simple models. This is likely the case also at the other planetary magnetospheres, where ULF waves have been studied but often only magnetic field measurements are available (e.g., James et al., 2016; Manners & Masters, 2019). These effects, however, do not appear to influence terrestrial magnetic perturbations measured from the ground since *∂h*/*∂s* has the same sign in both directions close to the Earth, and thus previous results from networks of ground magnetometers (e.g., Mathie et al., 1999; Samson et al., 1971; Ziesolleck & McDiarmid, 1995) remain reliable.

The example polarization ellipses shown in Figure 5b also indicate that the orientation of magnetic perturbations can differ to those in the velocity too. While the two are somewhat similarly oriented on the nightside, we find that on the dayside the magnetic field semi‐major axes tend be predominantly radial in orientation, differing from the velocity by ∼50–90° (apart from around noon where both quantities are radially aligned). The fact that the magnetic field's polarization can have a different orientation has not been stressed in the previous literature, since such studies have largely focused on either the displacement (Singer et al., 1981) or electric field (Degeling et al., 2014; Kabin et al., 2007; Rankin et al., 2006).

### 3.3 Standing Wave Detection for Spacecraft

Section 3.1 detailed the presence of standing structure spatially within the simulation, both across the geomagnetic field and along it. Outside of a simulation though, it is generally not possible to infer this due to spacecraft observations being sparse and subject to spatio‐temporal ambiguity. One common method of detecting standing waves is derived from the wave Poynting flux

(8) $$\begin{array}{ccc} S & = & \delta E\times \delta B/\mu_{0}\\ & = & \left(B_{0}\times \delta v\right)\times \delta B/\mu_{0}\\ & = & \left[-\left(\delta B_{⊥}\cdot \delta v_{⊥}\right)B_{0}+B_{0}\delta B_{∥}\delta v_{⊥}\right]/\mu_{0} \end{array}$$

and requiring the net energy propagation averaged over a cycle be zero in the direction the wave is standing (Kokubun et al., 1977). Using phasor notation, where instantaneous fields go as $\delta v\left(t\right)=\delta \tilde{v}e^{i\omega t}$ with the tilde indicating the phasor (complex amplitude), the complex Poynting vector can be constructed as

(9) $$\begin{array}{ccc} \tilde{S} & = & \delta \tilde{E}\times \delta {\tilde{B}}^{*}/2\mu_{0}\\ & = & \left[-\left(\delta {\tilde{B}}_{⊥}^{*}\cdot \delta {\tilde{v}}_{⊥}\right)B_{0}+B_{0}\delta {\tilde{B}}_{∥}^{*}\delta {\tilde{v}}_{⊥}\right]/2\mu_{0} \end{array}$$

The time‐averaged power flow is then simply given by $Re\left\{\tilde{S}\right\}$. In contrast, $Im\left\{\tilde{S}\right\}$ corresponds to reactive power—the flow of trapped energy that converts between electric and magnetic components without contributing to the propagation of the field—which can indicate the presence of standing waves. From Equation 9, we see that standing waves either parallel or perpendicular to the field have ±90° cross‐phases between components of the velocity and magnetic fields. Methods for detecting standing waves in spacecraft data thus search for this desired cross‐phase, though often make assumptions (based on simplified models) about the orientation of the wave perturbations.

#### 3.3.1 Standing Structure Across the Field

Nodal structure across the field on the nightside, which indicates the presence of waveguide modes, was commented on in Section 3.1.1. Waters et al. (2002) suggested such modes could be found where the compressional magnetic field and azimuthal electric field (equivalent to radial velocity) are in quadrature. This assumes the fast magnetosonic waves are standing in the radial direction, which would have been the case in the 3D wave simulation used by the authors (that of Lee & Lysak, 1999) since this has a dipole magnetic field and axially symmetric Alfvén speeds. This criterion has been used to detect such modes around noon in global MHD simulations (Hartinger et al., 2014) as well as in spacecraft observations (Hartinger et al., 2012, 2013). However, we find in our simulation that most of the regions that show clear evidence of monochromatic cavity/waveguide modes do not exhibit this required phase difference. This is likely because in a realistic magnetosphere fast waves do not necessarily interfere in the purely radial direction, though it is not clear which is the most physically appropriate direction to consider.

Wright and Elsden (2020) posed a different diagnostic for standing or propagating fast waves by explicitly calculating the right‐hand side of Equation 5 in their dipole field simulations with more realistic (non‐axially symmetric) Alfvén speeds. They showed through visual inspection that along a path parallel to the magnetopause, this was in‐phase with the compressional magnetic field around noon (indicating standing waves), become more ambiguous, and then was in quadrature at the distant flank (propagating waves). This method, however, cannot be applied to spacecraft data since it requires multipoint measurements for the calculation of derivatives as well as knowledge of the magnetic field geometry.

**Figure 7:**
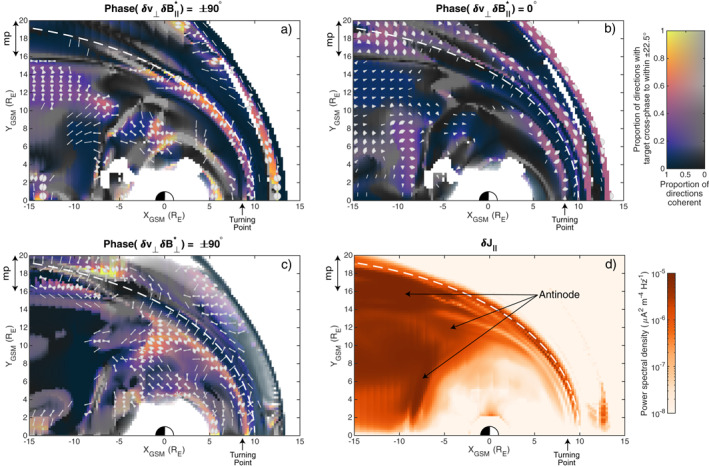
Projection in the *Z*_*GSM*_ = −2 *R*_*E*_ plane of directions perpendicular to the magnetic field (gray markers) with phase relations relevant for (a) standing structure across the field, (b) propagating structure across the field, and (c) standing structure along the field. Colors show the proportion of directions the phase relation is satisfied, with greys and whites indicating low coherence and power respectively. Wave power spectral density in the field‐aligned current is also shown (d).

Instead, we seek to generalize the Waters et al. (2002) criterion. Rather than only using the radial direction, we consider all directions perpendicular to the background field. For each one we calculate the cross‐phase between that component of *δ* **v**_⊥_ and the compressional magnetic field as well as their coherence. We then find in which directions the desired cross‐phase holds to within ±22.5° (45° bins centered on the target), is coherent (>0.8), and has significant wave power. The results are depicted in Figures 7a and 7b for standing (quadrature) and propagating (in‐phase) waves respectively. In these panels, colored regions indicate the desired cross‐phase is present in some direction, whereas blacks show this did not occur and greys depict a lack of coherence. The markers indicate the directions which satisfied all our criteria.

Figure 7a reveals a number of regions with standing structure across the magnetospheric magnetic field. The first of these is within the magnetopause current layer itself, spanning most of the dayside. Here the components of the velocity nearly tangent to the boundary are in quadrature with the compressional magnetic field. The second standing region is in the outer magnetospheric flanks (*X*_*GSM*_ < −5 *R*_*E*_), corresponding well with the location of the nodal structure in *δB*_∥_ identified previously. While far into the tail the standing directions are approximately aligned with ±*Y*_*GSM*_ it is clear that nearer Earth the standing axis tilts toward the Sun‐Earth line. The Earthward edge of the standing region approximately follows the contour of the Alfvén speed (Figure 2a), suggesting this constrains the penetration of fast magnetosonic waves from the magnetopause, in line with simple theory (Kivelson & Southwood, 1985; Kivelson et al., 1984). However, standing behavior is also present within the region of high Alfvén speed near the Earth (centered at *X*_*GSM*_ = −2 *R*_*E*_ and *Y*_*GSM*_ = 8 *R*_*E*_) which does not extend to the magnetopause. Curiously the standing direction changes considerably within this region, varying from quasi‐radial to almost azimuthal. Such behavior is not anticipated from models with axial symmetry and thus is likely due to wave refraction in the more realistic wave speed profile (Elsden & Wright, 2019; Wright et al., 2018). Finally, we note that within the dayside magnetosphere, where the surface mode dominates, quadrature emerges only deeper into the magnetosphere. This corresponds to where the effects of plasma compressibility become more negligible due to higher Alfvén speeds (Equation 1) and thus more evanescent and less propagating behavior is expected, as mentioned by A21.

We compare these results to Figure 7b, which indicates where and in which direction waves propagating across the field are present. Note that colors here are less intense since there is only one target cross‐phase value, whereas for standing waves there were two. In most regions, the direction of propagation is almost perpendicular to that in panel a, that is, the waves propagate transverse to the direction in which they are standing. The direction of propagation throughout is largely in line with the time‐averaged Poynting vector presented by A21—toward the subsolar point on the dayside and generally tailward on the nightside.

While we could have simply used the reactive power component of the complex Poynting vector, we note this requires considerable care. First, $Im\left\{{\tilde{S}}_{⊥}\right\}$ will only yield one direction, thus in cases where waves are standing in several directions $Im\left\{{\tilde{S}}_{⊥}\right\}$ will vary with location (true of even simple examples of a homogeneous rectangular cavity) thus does not always indicate the direction in which a wave is standing. Second, $Im\left\{{\tilde{S}}_{⊥}\right\}$ can have significant components along or anti‐parallel to the time‐averaged Poynting vector. These are clearly unrelated to a purely standing wave and may be the result of either the interference of waves of different amplitudes, near‐field antenna effects close to a current source, or interactions between the wave fields and the plasma. Finally, while $Im\left\{{\tilde{S}}_{⊥}\right\}$ can always be computed, it is necessary to determine whether the resulting vector (or a particular component) is statistically significant and thus meaningful, especially in the presence of background noise.

#### 3.3.2 Standing Structure Along the Field

To detect standing structure along the field, a criterion often used is a ±90° phase difference between a component of *δ* **B**_⊥_ and the same component of *δ* **v**_⊥_ (or equivalently the component of *δ* **E**_⊥_ perpendicular to both that direction and the background magnetic field; e.g., Kokubun et al., 1977; Takahashi & McPherron, 1984). This is generally referred to as a test for standing Alfvén waves and typically the radial or azimuthal directions are chosen. However, similarly to before, we generalize this criterion to consider all directions perpendicular to the background field in search of standing structure along it. The results are shown in Figure 7c in the same format as before.

Across most of the dayside magnetosphere evidence of standing structure along the field is present, in line with the results presented earlier. This is found mostly in the azimuthal direction, likely because the geometric effects pertaining to the radial direction lead to a different phase relationship than that predicted within a box or symmetric dipole model. However, we know that the signatures on the dayside cannot be explained as Alfvén waves because the observed frequency is too low and the mode of oscillation is primarily compressional. Additional evidence against a pure Alfvén mode is also given in Figure 7d, which shows a power map (similar to those in Figure 2) for the field‐aligned current *δJ*_∥_. While Alfvén waves are associated with such currents, fast magnetosonic waves (either propagating or evanescent) are generally not (e.g., Wright & Elsden, 2020). Indeed, *δJ*_∥_ is weak throughout the dayside magnetosphere. Therefore, we stress that a ±90° phase difference between *δ* **B**_⊥_ and *δ* **v**_⊥_ does not necessarily indicate a standing Alfvén wave, but simply standing structure along the field. There are, however, strong field‐aligned currents within the magnetopause boundary layer. This agrees with theoretical predictions for surface eigenmodes in a box model, which are supported by currents flowing entirely within the boundary that are closed via the ionospheres (Plaschke & Glassmeier, 2011).

On the nightside, in general there is little standing structure along the field present as evident in Figure 7c. This may be due to the nightside field lines being much longer, and thus having eigenfrequencies much lower than 1.8 mHz, or indeed some field lines not even being closed within the full simulation domain. We do, however, observe a few areas of localized standing structure present, for example, at around *X*_*GSM*_ = −8 *R*_*E*_ near the central magnetotail as well as along *Y*_*GSM*_ = 11 *R*_*E*_ near the terminator. These regions correspond well with the subtle antinodes in *δ* **v**_⊥_ identified in Figure 2b and also exhibit enhanced field‐aligned currents, as seen in Figure 7d. Therefore, we conclude that these do in fact correspond to Alfvén modes.

Similarly to across the field, we find that if only the usual radial/azimuthal directions are used then most areas with standing structure along the field are missed. This again highlights the need to consider all directions perpendicular to the field when in a realistic magnetosphere. We also note that an alternative test requiring significant reactive power along the field, $Im\left\{{\tilde{S}}_{∥}\right\}$, could be used though the same care to that outlined earlier is required.

## 4 Summary

We have investigated the polarizations of system‐scale MHD waves in a realistic magnetosphere through using a global MHD simulation of the resonant response to a solar wind pressure pulse. While many aspects of the surface, fast magnetosonic, and Alfvén waves excited are in agreement with simple box models, we find that some of the predictions are significantly altered. The key findings are the following:

1. The often assumed order from the fast magnetosonic dispersion relation of a turning point followed by matching Alfvén resonant location does not always hold for radially monotonic Alfvén speed profiles. This prediction arises from box models with inhomogeneity only in the radial direction. Southwood and Kivelson (1986) showed introducing additional inhomogeneity along the field within such a model allows fast mode waves to drive field line resonances exterior to their turning points, that is, in the region where they are propagating. Here, we find a similar effect for magnetopause surface modes. The location in which the surface wave frequency matches the Alfvén mode occurs within the current layer, as previously suggested by Kozyreva et al. (2019). The turning point of the wave, beyond which it becomes evanescent, however, occurs outside of the boundary layer itself within the magnetosphere.
2. Realistic magnetic geometries introduce additional nodes to perpendicular magnetic field oscillations that are not present in the velocity. The nodes occur at the apexes of field lines, particularly near the cusps, since large‐scale radial displacements of the field line at these locations will not cause deflection of the magnetic field vectors. These effects are not present in box models since they contain no such apexes as field lines are straight.
3. A reversal in the compressional magnetic field occurs at high latitudes near the cusps due to the non‐uniform magnetic field. This occurs even when the plasma displacement has fundamental standing structure along the field and thus nodes only at the ionospheres. We show that both dipole and global MHD magnetic fields predict such a reversal of the compressive oscillations due to the gradients of scale factors and field strengths present. Only waves with short radial extents (≪1–2 *R*_*E*_) are likely unaffected.
4. We report on the velocity polarizations associated with stationary surface waves subject to a non‐zero magnetosheath flow. For zero external flow, no handedness is predicted since perturbations are a simple breathing motion back and forth (Lamb, 1895). Stationary waves are possible under non‐zero magnetosheath flows via the surface wave propagating against the flow, balancing its advective effect (A21). But surface waves transfer momentum to particles in the direction of propagation, causing them to undergo cycloidal motion (Stokes, 1847). This, therefore, results in a handedness to the polarization—right‐handed with respect to the field in the pre‐noon sector and left‐handed post‐noon. Once advection overcomes the wave propagation sweeping the waves tailward, outside of the 09 < MLT < 15 hr range, the usual sense of polarization is recovered (Samson et al., 1971; Southwood, 1968). Therefore, velocity (or equivalently electric field) polarizations measured by spacecraft may be a useful technique in detecting MSEs.
5. In the outer magnetosphere, close to the magnetopause, the polarization of the magnetic field has opposite handedness to that of the velocity due to geometric effects of the cusps. Local maxima in the radial scale factors occur away from the magnetic equator and toward the cusps for these field lines, unlike in a dipole field line. However, azimuthal scale factors still decrease away from the equator. Therefore, the gradient along the field of the scale factors is opposite for the two directions between these local maxima. This results (in the long wavelength limit applicable here) in the observed opposite handedness of the magnetic field. Polarizations measured from the ground, however, are not affected and thus they may be used in diagnosing magnetospheric normal modes.
6. Widely used detection techniques for standing structure both across (Waters et al., 2002) and along (Kokubun et al., 1977; Takahashi & McPherron, 1984) the field can fail in a realistic magnetosphere. These make assumptions, based on axially symmetric models, on the directions in which to compute cross‐phases between quantities. We show that in a realistic magnetosphere they are not always the appropriate directions to use and that a method that considers all directions perpendicular to the background magnetic field is required.

While we have only focused on one frequency range within this simulation, we conclude that these effects occur when the characteristic spatial scales of waves are much longer than those of changes in the geometry or magnetic field. Eigenfrequencies of the MHD wave modes depend on the Alfvén speeds throughout the system, thus for different conditions similarly large‐scale (>2 *R*_*E*_) waves will occupy different frequency ranges (Archer & Plaschke, 2015; Archer et al., 2015, 2017). Therefore, our results should be applicable beyond simply the frequency range presented.

Fully exploring the implications of these results on energy transfer throughout geospace warrants dedicated study, though we briefly discuss their possible impacts. It is clear from Equation 9 that the changes to the surface mode's magnetic field perturbations introduced at high latitudes affect the waves' energy flux. This should be most significant along the field, likely increasing dissipation both in the boundary layer (Chen & Hasegawa, 1974) and ionosphere (Allan, 1982; Southwood & Kivelson, 2000). Additionally, our results suggest bouncing radiation belt particles are subject to more compressional wave power, at high latitudes, than would be expected from box models. This could lead to enhanced radial diffusion (e.g., Elkington, 2006).

The simulation presented here offers a more representative magnetosphere than box or axially symmetric dipole models of ULF waves. However, there are further improvements that could make the magnetosphere even more realistic. First, the run presented is perfectly North‐South and dawn‐dusk symmetric due to the use of a fixed dipole with zero tilt, no plasma corotation, and perfectly northward IMF. Second, the uniform ionospheric conductivity used is unrealistic and could be improved to include the auroral oval and inter‐hemispheric differences (e.g., Ridley et al., 2004, 2006). The introduction of asymmetries to the system could thus be studied.

No plasmasphere or ring current was included, since our focus was the outer magnetosphere. These would lead to non‐monotonic wave speed profiles that might enable fast magnetosonic waves to penetrate the magnetosphere more deeply (e.g., Claudepierre et al., 2016) as well as introduce the possibility of plasmaspheric cavity modes (e.g., Waters et al., 2000), neither of which should affect our conclusions.

Finally, we note the version of BATS‐R‐US uses an isotropic pressure. This can cause unphysical mixing in collisionless space plasmas between parallel and perpendicular pressures, affecting the magnetosonic wave modes. While low‐*β* magnetospheric plasmas will be little affected, and the results presented have all been reconciled with theory, there may in reality be differences in high‐*β* areas such as the magnetosheath or cusps. Incorporating pressure anisotropy could be investigated, though the typical Chew‐Golderberger‐Low (CGL) kinetic approximation of the MHD equations often applied to simulations (Chew et al., 1956; Meng et al., 2012) might not be appropriate for the low frequencies under consideration since the accessible volume to particles becomes essentially the entire flux tubes. It is conceivable that appropriately modeling the cusps though might reveal a hitherto unforeseen eigenmode corresponding to magnetosonic (or sound) waves trapped within the cone‐like cavity of each magnetospheric cusp, a challenge we leave to future work.

**Notation**
*msh*
: Magnetosheath
*mp*
: Magnetopause
*sph*
: Magnetosphere
*GSM*
: Geocentric Solar Magnetospheric coordinates
∥: Field‐aligned
*α*
: Arbitrary perpendicular coordinate
*β*
: Arbitrary perpendicular coordinate
*ϵ*
: Ellipticity
*θ*
: Colatitude
*ϕ*
: Azimuthal angle
*ϕ*_⊥_
: Perpendicular azimuthal coordinate
*μ*_0_
: Vacuum permeability
*ξ*
: Plasma displacement
*ρ*
: Mass density
*ω*
: Angular frequency
**B**
: Magnetic field
*c*_*s*_
: Speed of sound
**e**
: Orthonormal basis vector
**E**
: Electric field
*h*
: Curvilinear scale factor
**J**
: Current density
**k**
: Wave vector
*r*_⊥_
: Perpendicular radial coordinate
**r**
: Geocentric Position
*s*
: Field line length
**S**
: Poynting vector
*t*
: Time
**v**
: Plasma velocity
*v*_*A*_
: Alfvén speed

## Supporting information

Supporting Information S1

Movie S1

## Data Availability

Simulation results have been provided by the Community Coordinated Modeling Center (CCMC) at Goddard Space Flight Center using the SWMF and BATS‐R‐US tools developed at the University of Michigan's Center for Space Environment Modeling (CSEM). This data are available at https://ccmc.gsfc.nasa.gov/results/viewrun.php?domain=GM&runnumber=Michael_Hartinger_061418_1.

## Appendix A: Dipole Magnetic Field

In a dipole magnetic field, field lines for a given *L*‐shell are given by

(A1)
$$r=L\sin^{2}\theta$$

where *r* is the geocentric distance and *θ* the colatitude. Their length is (Chapman & Sugiura, 1956)

(A2)
$$s=\frac{2L}{\sqrt{3}}\left\{\sinh^{-1}\left[\sqrt{3\left(1-\frac{1}{L}\right)}\right]+\sqrt{3\left(1-\frac{1}{L}\right)\left(4-\frac{3}{L}\right)}\right\}$$

and the field strength is

(A3)
$$B_{0}=B_{E}{\left(\frac{R_{E}}{r}\right)}^{3}\sqrt{1+3\cos^{2}\theta }$$

where *B*_*E*_ = 3.12 × 10^−5^ T is its equatorial value on Earth's surface. One possible scheme of dipole coordinates uses perpendicular radial and azimuthal coordinates (and corresponding scale factors) given by (Swisdak, 2006; Wright & Elsden, 2020)

(A4)
$$\begin{array}{cc} \begin{array}{ccc} r_{⊥} & = & \frac{r}{\sin^{2}\theta }\\ h_{r_{⊥}} & = & \frac{\sin^{3}\theta }{\sqrt{1+3\cos^{2}\theta }} \end{array} & \begin{array}{ccc} \phi_{⊥} & = & \phi \\ h_{\phi_{⊥}} & = & r\sin\theta \end{array} \end{array}$$

From these definitions, it can be shown that

(A5)
$$\frac{\partial h_{\phi_{⊥}}}{\partial r_{⊥}}=\frac{\partial h_{\phi_{⊥}}}{\partial r}{\left(\frac{\partial r_{⊥}}{\partial r}\right)}^{-1}+\frac{\partial h_{\phi_{⊥}}}{\partial \theta }{\left(\frac{\partial r_{⊥}}{\partial \theta }\right)}^{-1}=\frac{1}{2}\sin^{3}\theta$$

and

(A6)
$$\frac{\partial B_{0}}{\partial r_{⊥}}=\frac{\partial B_{0}}{\partial r}{\left(\frac{\partial r_{⊥}}{\partial r}\right)}^{-1}+\frac{\partial B_{0}}{\partial \theta }{\left(\frac{\partial r_{⊥}}{\partial \theta }\right)}^{-1}=-\frac{12B_{0}}{r}\frac{\cos^{2}\theta }{1+3\cos^{2}\theta }$$

## Appendix B: Polarization Parameters

Parameters of the polarization ellipse are determined from power spectra (Arthur et al., 1976; Collett, 2005) as detailed here. First, the auxiliary angle *η* is calculated via

(B1)
$$\tan\eta =\sqrt{\frac{P_{yy}}{P_{xx}}}$$

where *P* represents the power spectral density matrix with *x* and *y* being two orthogonal directions that are both perpendicular to the background magnetic field. Combining this with the cross‐phase Δ, the phase difference between the oscillations in the *y* and *x* directions, gives the ellipticity

(B2)
$$\epsilon =\frac{\sin2\eta \cdot \sin\Delta }{2\sqrt{1-1/4\sin^{2}2\eta \cdot \sin^{2}\Delta }}$$

which has values between −1 (right‐hand circularly polarized) and +1 (left‐hand circularly polarized). The orientation angle *ψ* that the semi‐major axis makes with the *x* direction is given by

(B3)
sin2ψ=tan2η⋅sinΔ

We also calculate the four Stokes (1852) parameters

(B4)
$$\begin{array}{ccc} S_{0} & = & P_{xx}+P_{yy}\\ S_{1} & = & P_{xx}-P_{yy}\\ S_{2} & = & 2Re\left\{P_{xy}\right\}\\ S_{3} & = & -2Im\left\{P_{xy}\right\} \end{array}$$

where *S*_0_ is the total variance, *S*_1_ and *S*_2_ correspond to linearly polarized waves, and *S*_3_ to circularly polarized waves (Collett, 2005). The degree of polarization *p* is thus

(B5)
$$p=\frac{\sqrt{S_{1}^{2}+S_{2}^{2}+S_{3}^{2}}}{S_{0}}$$

which varies between 0 (unpolarized) and 1 (fully polarized).
