# Supplementary material for: How a Realistic Magnetosphere Alters the Polarizations of Surface, Fast Magnetosonic, and Alfvén Waves
Source: J Geophys Res Space Phys. 2022 Feb 7;127(2):e2021JA030032. doi: 10.1029/2021JA030032 (PMC9286832; doi:10.1029/2021JA030032)
Supplement: Supplementary file 1 — Supporting Information S1 [file JGRA-127-0-s002.pdf]

Supporting Information for "How a realistic magnetosphere alters the polarizations of surface, fast magnetosonic, and Alfvén waves"

M. O. Archer, [1]

D. J. Southwood, [1]

M. D. Hartinger, [2]

L. Rastaetter, [3]

and A. N. Wright [4]

[1]Space and Atmospheric Physics Group, Department of Physics, Imperial College London, London, UK.

[2]Space Science Institute, Boulder, Colorado, USA.

[3]NASA Goddard Space Flight Center, Greenbelt, Maryland, USA.

[4]Department of Mathematics and Statistics, University of St Andrews, St Andrews, UK.

Contents of this file

1. Table S1

Additional Supporting Information (Files uploaded separately)

1. Captions for Movie S1

Introduction

This supporting information provides a table detailing the simulation run and a movie of the simulation output.

Movie S1.

Movie of the global MHD in the GSM XZ plane depicting, through the colour scales, filtered perturbations in the compressional magnetic field (left), perpendicular radial velocity (middle), and field-aligned velocity (right) components. The magnetopause location (black) is indicated, identified as the last closed field line using a bisection method, and field line tracings are also shown.

**Simulation Setup**		
Dipole GSM Orientation		(0,0,1)
Dipole Update		No

**Solar Wind Conditions**		
	**Solar Wind**	**1 min Density Pulse**
n (cm^{-3})	6	14
T (K)	116,174.0	49,788.8
$\mathbf{v}_{GSM}$ ($\mathrm{km\,s}^{-1}$)	(-450,0,0)	(-450,0,0)
$\mathbf{B}_{GSM}$ (nT)	(0,0,5)	(0,0,5)
GSM normal		(1,0,0)

**Ionospheric Conductivity**		
Uniform		5 mho

Table S1. Details of the SWMF global MHD simulation run used in this paper.
